# Efficacy and safety of traditional chinese medicine treatment for overweight and obese individuals: A systematic review and meta-analysis

**DOI:** 10.3389/fphar.2022.964495

**Published:** 2022-10-05

**Authors:** Zhi Ge Wen, Qi Qi Zhang, Li Li Zhang, Meng Fei Shen, Yi Shan Huang, Lin Hua Zhao

**Affiliations:** ^1^ Institute of Metabolic Diseases, Guang’anmen Hospital, China Academy of Chinese Medical Sciences, Beijing, China; ^2^ Graduate School, Beijing University of Chinese Medicine, Beijing, China

**Keywords:** traditional Chinese medicine, obesity, overweight, systematic review, meta-analysis

## Abstract

**Background:** The prevalence of obesity is increasing worldwide, causing a global health issue. Traditional Chinese medicine (TCM) used in treating overweight/obesity has been widely implemented in clinical practice, but its overall efficacy and safety remain unclear. This review aims to evaluate the effectiveness and safety of TCM based on randomized controlled trials (RCTs).

**Methods:** A systematic review was conducted by searching PubMed, Cochrane Library, Web of Science, Embase, and Clinical Trails from their inception to March 2021. Two reviewers screened studies, extracted the data, and assessed the risk of bias independently. The data were pooled for meta-analysis or presented narratively.

**Results:** Twenty-five RCTs involving 1,947 participants were included. Compared with placebo or blank control, TCM preparations reduced Body Mass Index (BMI) [MD = −1.16; 95% confidence interval (CI) = −1.44, −0.89; I^2^ = 34%], reduced weight (MD = −2.53; 95% CI = −3.08, −1.99; I^2^ = 34%), reduced waist circumference (MD = −2.64; 95% CI = −3.42, −1.87; I^2^ = 0%), reduced hip circumference (MD = −3.48; 95% CI = −4.13, −2.83; I^2^ = 0%), reduced total cholesterol (TCHO) (MD = −10.45; 95% CI = −18.92, −1.98; I^2^ = 63%), reduced triglycerides (TG) (MD = −4.19; 95% CI = −6.35, −2.03; I^2^ = 25%), increased high-density lipoprotein (HDL) (MD = −3.60; 95% CI = −6.73, −0.47; I^2^ = 81%), reduced fasting blood glucose (FBG) (MD = −0.77; 95% CI = −1.24, −0.29; I^2^ = 91%). Glycated hemoglobin (HbA1c)、body fat rate、low-density lipoprotein (LDL) were not statistically significant. For people with hypertension, decreased systolic blood pressure (SBP) (MD = −5.27; 95% CI = −8.35, −2.19; I^2^ = 58%), decreased diastolic blood pressure (DBP) (MD = −4.30; 95% CI = −5.90, −2.69; I^2^ = 0%). For people with normal blood pressure, there was no significant change. There was no significant difference in liver function.

**Conclusion:** It has been demonstrated that TCM preparations have good clinical efficacy and safety for overweight/obesity. TCM may be suitable for overweight/obesity in adult populations for its efficacy and safety of long-term treatment.

## 1 Introduction

Over the past 50 years or so, the prevalence of overweight and obesity has increased globally, reaching pandemic levels (Bluher, 2019). The number of cases of obesity and related diseases has significantly increased globally. By 2019, there were more than 1.9 billion overweight adults and 650 million obese adults (Yao et al., 2017). Obesity is one of the leading preventable threats to global health. Excessive obesity is defined as the excessive accumulation of fat in adipose tissue due to an imbalance between energy intake and energy expenditure (Yao et al., 2017). Obesity is a major health challenge and a negative factor affecting the health and longevity of community residents. Obesity is associated with many diseases, including cardiovascular diseases, diabetes mellitus, hypertension, hyperlipidemia, and fatty liver (Ogden et al., 2014).

Weight control can alleviate these problems (Monteiro and Azevedo, 2010). Current research has proved that lifestyle interventions for obese adults were effective in reducing weight (5% or more of initial weight) and the incidence rate of diabetes (Lee and Cha, 2016; Ryan and Yockey, 2017). For other related health problems, such as cardiometabolic risk factors, the benefits are unclear. Drug therapy is used as an adjunct to lifestyle, especially when lifestyle changes fail to produce an ideal weight loss effect, and the choice of drugs depends on the presence of comorbidities (Fisher et al., 2018). The guidelines suggested that patients with body mass index (BMI) > 27 kg/m^2^ and obesity-related complications or BMI >30 kg/m^2^ can take orally five kinds of weight loss drugs approved by the United States Food and Drug Administration (Jackson et al., 2015): orlistat, liraglutide, clokaserin, naltrexone/amphetamine compound, and fentamine/topiramate compound. At present, surgery has also been proven to be effective for severely obese people (Apovian et al., 2015; LeBlanc et al., 2018). Bariatric surgery can reduce the risk of obesity-related complications, but its significant costs and risks severely limit its widespread use (Waters et al., 2013). Up to no, obesity prevention and treatment strategies have failed to achieve long-term success either at the individual or group level.

In China, the obese population is also growing, and health problems are becoming more prominent. TCM has a long history of thousands of years. It is widely used to treat a variety of chronic diseases, including overweight, obesity, and these complications. TCM can play an important role in the treatment of obesity (Kazemipoor et al., 2015; Sahebkar-Khorasani et al., 2019) by inhibiting appetite (Pories, 2008), stimulating thermogenic metabolism promoters, inhibiting pancreatic lipase activity, reducing fat absorption, increasing fat decomposition and reducing fat production (Hong et al., 2017; Liu et al., 2019). However, conflicting opinions still exist due to the lack of sufficient evidence to support the efficacy and safety of TCM for the treatment of overweight and obesity. For these reasons, RCTs have also been conducted to evaluate the scientific evidence on the effectiveness of TCM. This paper systematically reviews the potential role of TCM in the treatment of overweight and obesity and summarizes the scientific evidence.

The previous systematic review evaluated the efficacy of TCM and its products in treating obesity and metabolic syndrome (Payab et al., 2020). In recent years, with the increasingly serious health risks brought by obesity, it is necessary to systematically evaluate the effectiveness and safety of only using TCM in overweight and obese people. Although not enough RCTs of TCM for overweight and obesity have been retrieved, and the population base of clinical trials is small, we try to provide the most authentic and reliable evidence on the effectiveness and safety of TCM for treating overweight and obesity.

## 2 Materials and methods

### 2.1 Search strategy

We comprehensively searched five English language databases, PubMed, Embase, Cochrane Library, Web of Science, and Clinical Trails, from inception to 15 March 2021. We used traditional Chinese medicine, Chinese medicine treatment of overweight or obesity, randomized controlled retrieval overweight or obesity, randomized controlled clinical trials, and meta-analyses as the keywords. Additional studies were searched in the reference lists of all identified publications, including relevant meta-analysis and systematic reviews. Finally, we identified 25 published randomized controlled clinical trials that met the inclusion criteria.

### 2.2 Inclusion criteria

We included all published RCTs and met the following criteria: 1) Participants were overweight or obese adults (age ≥ 18 years, BMI ≥ 24 kg/m^2^). 2) With or without other obesity-related metabolic diseases. 3) RCTs. 4) Control was placebo or blank. 5) The treatment group used herbal preparations including a single botanical drug, poly-herb, or herbal extracts.

### 2.3 Exclusion criteria

We excluded the following characteristics of clinical studies: 1) Patients with drug-induced obesity, i.e., drug-induced obesity. 2) Patients taking hormones were excluded.

### 2.4 Data extraction

Two students independently extracted data from 25 original test reports using standardized forms. The extracted data include the characteristics of 25 clinical trials (first author, year of publication, sample size, intervention and control, treatment cycle, and follow-up time), characteristics of 1,947 patients (inclusion criteria, average age, male proportion, intervention and control measures, baseline weight, waist circumference, BMI, waist circumference, hip circumference, FBG, blood pressure, blood lipid level and safety index level), outcome BMI, weight, waist circumference, waist circumference, hip circumference, and FBG, blood pressure, blood lipid levels, safety indicators, and adverse events) and methodological information. When we needed additional information that was not available in online publications or supplementary materials, we contacted the study authors.

### 2.5 Quality assessment

We used the Cochrane bias risk tool to assess the bias risk of RCTs (Higgins et al., 2019). Two investigators independently completed the assessments, and discrepancies were discussed with a third person and resolved by consensus. Additionally, the GRADE (Grading of Recommendations Assessment, Development, and Evaluation) framework was used to assess the quality of evidence contributing to each network estimate. This framework characterizes the quality of the body of evidence based on study limitations, imprecision, inconsistency, indirectness, and publication bias of the main results. (Guyatt et al., 2008).

### 2.6 Statistical analyses

The data entry and analysis were conducted using Microsoft Excel 2016 and Review Manager software version 5.3, respectively. The risk ratio and standard mean difference with a 95% confidence interval (CI) of the outcomes were calculated as the effective measures. We calculated the heterogeneity of the I^2^ statistic as a measure of the proportion of overall variation attributable to inter-study heterogeneity. The fixed-effects (FE) model was used if I^2^ < 50%; otherwise, the random-effects model was used. Additionally, sensitivity analyses were performed before combining RCTs in the meta-analyses to determine possible additional sources of heterogeneity and changes in effect sizes. Publication bias was tested by visual inspection of the funnel plots. When few studies are included in the analysis, the power of the tests is too low. Therefore, publication bias was only examined if > 10 study comparisons were included in the analysis (Bluher, 2019).

## 3 Results

### 3.1 Study characteristics

**FIGURE 1:**
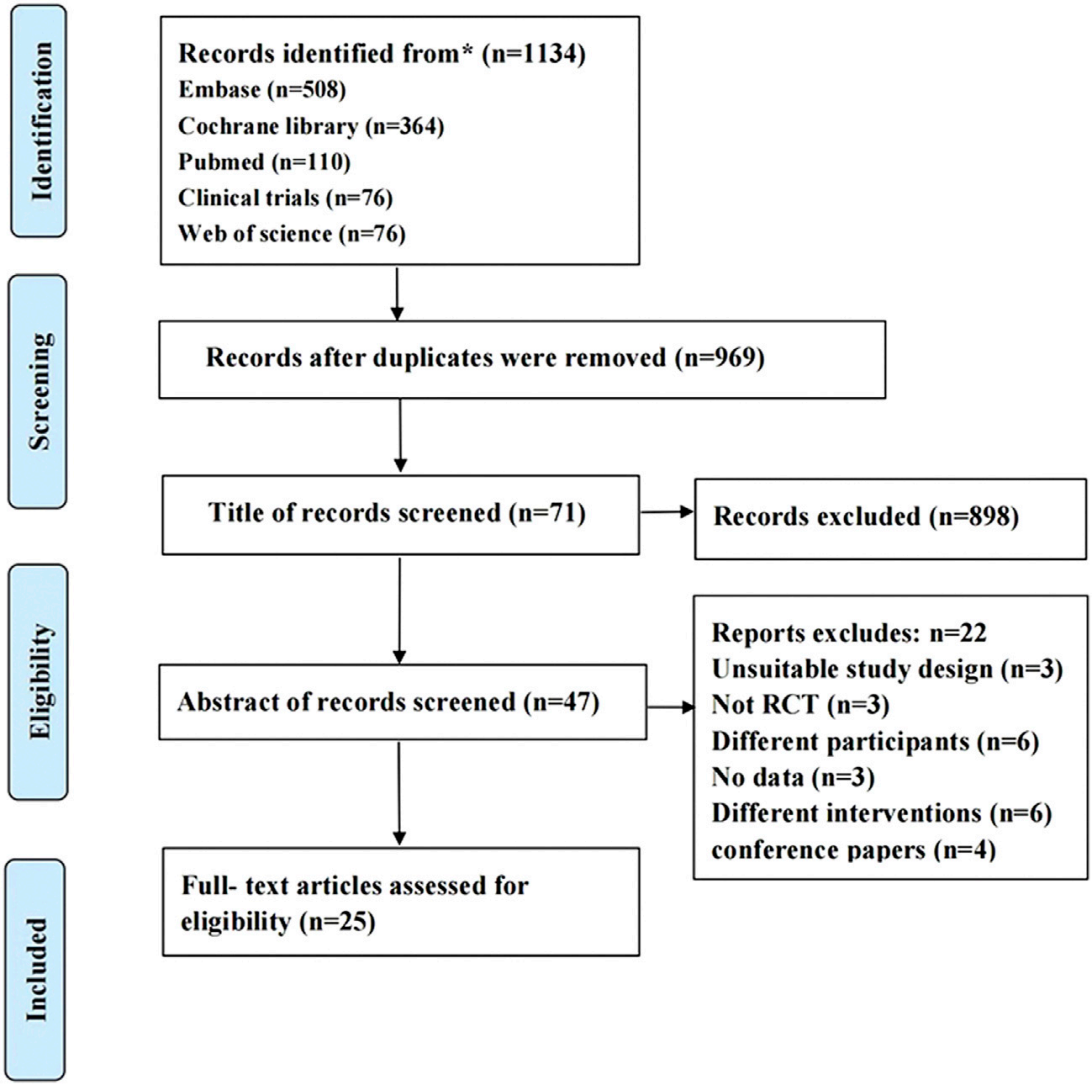
Prisivia flow chart of literature searching.

The search identified 1,143 papers, of which 165 were duplicates. Then, 71 articles remained after screening titles and abstracts, and 47 articles remained after the full-text screening. Finally, 25 eligible manuscripts (1,947 participants) (Boozer et al., 2001; Coffey et al., 2004; Hioki et al., 2004; Wang et al., 2006; Kim et al., 2008; Kamiya et al., 2011; Kamali et al., 2012; Ke et al., 2012; Lenon et al., 2012; Sengupta et al., 2012; Kazemipoor et al., 2013; Ofner et al., 2013; Park et al., 2013; Tong et al., 2013; Park et al., 2014; Azushima et al., 2015; Mirtaheri et al., 2015; Chung et al., 2016; Kudiganti et al., 2016; Satapathy et al., 2016; Cho et al., 2017; Zuniga et al., 2017; Daneshi-Maskooni et al., 2018; Gholaman and Gholami, 2018; Cheon et al., 2020) assessed the effect of TCM intervention on weight loss in overweight and obese people. The subjects were all overweight and obese people, of which 14 were simple overweight or obesity and the remaining 11 trials were overweight and obesity combined with one or more metabolic risk factors (e.g., abnormal blood lipids and blood pressure, or varying degrees of blood glucose abnormalities: impaired glucose tolerance or newly diagnosed patients with type 2 diabetes mellitus). Figure 1 shows the screening process. Table 1 shows the characteristics of included studies. Supplementary materials show the ingredients for each study herbal treatment group. According to the composition, TCM groups are divided into two subgroups, preparations based on a single botanical drug group and polyherbal preparations group.

**TABLE 1:** Characteristics of included studies.

Study ID	Sample size	Age (years old) (m ± sd)		Number (male/Total)		Base line BMI (m±sd)		Study population	intervention		Lifestyle intervention		Study duration
		TCM	Control	TCM	Contral	TCM	Contral		TCM	Contral	Diet	Sports	
Boozer et al., (2001)	67	40.0 ± 9.4	42.2 ± 8.1	4/32	6/35	32.6 ± 2.9	32.7 ± 2.7	simple obesity	Ma Huang-Guarana combination, 2 tablets, three times daily	placebo, 2 tablets, three times daily	limit intake of dietary fat to 30% of calories	walking 30 min a day, three times a week	8 weeks
Coffey et al., (2004)	102	44.9 ± 9.1	42.1 ± 10.9	5/52	9/50	35.1 ± 2.9	34.0 ± 2.9	simple obesity	125 mg Ma huang, 250 mg Kola nut, and 100 mg White willow bark, two caplets, three times daily	Placebo, two caplets, three times daily	a pamphlets about lifestyle modifications but no additional counseling		12 weeks
Hioki et al., (2004)	81	52.6 ± 14.0	54.8 ± 12.5	0/41	0/40	36.7 ± 6.80	36.1 ± 3.30	with Impaird glucose tolerance (IGT)	Bofu-tsusho-san, three times daily	placebo, three times daily	1,200 kcal a day for the two months before the start of the study	5,000 steps a day for the two months before the start of the study	24 weeks
Wang et al., (2006)	60	50.97 ± 11.10	49.24 ± 10.07	19/31	18/29	28.02 ± 2.17	28.72 ± 2.23	with hypertension	Pinggan Yishen Ditan Yin, take one dose a day, twice; lotensin, 10 mg a day	lotensin, 10 mg a day	reasonable diet	moderate exercise	8 weeks
Kim et al., (2008)	37	33.8 ± 7.9	30.8 ± 7.4	0/21	0/16	27.4 ± 2.3	27.9 ± 2.0	simple overweight/obesity	2 g of ephedra and 1 g corn starch	placebo, 3 g corn starch	low-calorie diet of 1,200 kcal a day	40 min walk, five times in a week	8 weeks
Kamiya et al., (2011)	36	39.3 ± 12.4	36.7 ± 9.4	15/18	15/18	25.5 ± 2.9	26.5 ± 2.5	simple overweight/obesity	300 mg PFE	300 mg placebo	males: 2,650 kcal a day females: 2,300 kcal a day	NA	8 weeks
Kamali et al., (2012)	60	39.16 ± 9. 59	36.36 ± 9. 9	6/30	8/30	37.14 ± 5.40	36.29 ± 4.66	simple obesity	Itrifal Saghir, 5 g, twice daily	placebo, 5 g, twice daily	keep existing diet and life style during the study period		12 weeks
Ke et al., (2012)	85	46.5 ± 7.3	45.7 ± 7.5	23/45	20/40	28.7 ± 3.4	28.5 ± 3.7	with IGT	Linggui Zhugan Decoction, twice a day for a month, Stop the medicine for one month, three consecutive cycles	blank contral	carbohydrates (50%-60%), protein ≤ 30%, high-fiber; proportions of three meals, 2:2:1	60 min each time, three times a week, six months.	6 months
Sengupta et al., (2012)	41	41.6 ± 1.37	37.2 ± 1.52	7/21	5/20	34.41 ± 0.74	33.0 ± 0.73	simple obesity	LI85008F, 900 mg a day	placebo, 900 mg a day	2,000 kcal standard diet	Walk 5 days a week, 30 minutes each time	8 weeks
Lenon et al.,(2012)	117	39.3 ± 13.2	40.4 ± 10.2	10/59	10/58	35.3 ± 4.8	36.0 ± 5.5	simple obesity	RCM-104, 4 capsules per time, three times per day	placebo, 4 capsules per time, three times per day	keep existing diet and life style during the study period		12 weeks
Tong et al., (2013)	399	54.4 ± 7.7	54.5 ± 7.6	149/292	47/107	26.3 ± 2.1	26.4 ± 2.4	with Type 2 diabetes mellitus (T2DM)	Tang-Min-Ling-Wan, 6 g, three times daily	placebo, 6 g, three times daily	NA	NA	12 weeks
Kazemipoor et al., (2013)	70	37.23 ± 9.34	37.00 ± 7.90	0/35	0/35	29.24 ± 3.36	30.39 ± 4.69	simple overweight/obesity	caraway seed extract, 30 ml, once daily	placebo, 30 ml, once daily	NA	aerobics training for 180 minutes a week	12 weeks
Park et al., (2013)	112	39.2 ± 9.5	38.8 ± 10.1	7/57	10/55	31.8 ± 2.6	31.9 ± 3.8	controlled hypertension or T2DM or treated hyperlipidemia	Taeeumjowi-tang, 7 g, three times daily	placebo, 7 g, three times daily	1,500 kcal a day for men and 1,200 kcal a day for women	NA	12 weeks
Ofner et al., (2013)	40	48.1 ± 9.6	46.8 ± 9.4	4/20	4/20	31.2 ± 4.7	30.0 ± 5.3	simple overweight/obesity	Salacia reticulata and vitamin D3 (SRD), three times daily	blank contral	guideline for lifestyle	twice-weekly training programs (each session 45 minutes)	4 weeks
Park et al., (2014)	111	41.56 ± 8.62	39.21 ± 10.12	unclear/55	unclear/56	29.72 ± 6.17	29.28 ± 3.11	simple overweight/obesity	Bofutsushosan, 12 capsules a day	placebo, 12 capsules a day	20-25 kcal a day per kg body weight	NA	8 weeks
Mirtaheri et al., (2015)	64	36.0 ± 11.97	33.6 ± 4.8	unclear/32	unclear/32	33.6 ± 4.8	32.7 ± 3.7	simple obesity	licorice extract, 0.5 g, three times daily	placebo, 0.5 g, three times daily	reduce energy intake by 500 kcal	NA	8 weeks
Azushima et al., (2015)	106	59.2 ± 14.5	60.0 ± 12.9	28/54	29/52	31.3 ± 5.0	30.6 ± 4.9	with hypertension	Bofu-tsusho-san, 2.5 g, once daily	conventional control therapy group, 2.5 g, once daily	25-30 kcal/kg-standard body weight a day	exercise therapies	24 weeks
Kudiganti et al., (2016)	60	36.63 ± 1.64	39.47 ± 1.73	10/30	14/30	28.48 ± 0.25	28.20 ± 0.24	simple obesity	Meratrim: one capsule (400 mg), two times daily	placebo, one capsule (400 mg), two times daily	2,000 kcal a day	30 min walk for five days per week	16 weeks
Satapathy et al., (2016)	30	21.06 ± 1.39	20.86 ± 0.66	unclear/16	unclear/14	25.21 (24.03–28.35)*	26.25 (24.49–27.70)*	simple overweight/obesity	Tulsi (Ocimum sanctum) extract: 250 mg, twice daily	blank contral	keep their diet and sports		8 weeks
Cho et al., (2017)	60	39.5 ± 11.2	41.7 ± 11.1	10/30	8/30	27.1 ± 1.5	27.2 ± 1.2	simple overweight/obesity	YY-312, 800 mg, three times daily	placebo, 800 mg, three times daily	reduce their energy intake by 500 kcal a day	maintain their usual level of physical activity	12 weeks
Chung et al., (2016)	20	50.00 ± 5.85	45.20 ± 9.52	6/10	6/10	29.50 ± 3.63	28.89 ± 2.96	more than 2 metabolic risk factors	Qingxue Dan, 900 mg a day	placebo, 900 mg a day	write a self-reporting diet and exercise diary everyday		8 weeks
Zuniga et al., (2017)	24	43 ± 1	41 ± 4	2/12	2/12	31.2 ± 2.5	30.3 ± 3.2	2 metabolic risk factors	Gymnema sylvestre, 300 mg, twice daily	placebo, 300 mg, twice daily	maintain their normal diet and physical activity levels		12 weeks
Daneshi-Maskooni et al., (2018)	20	unclear	unclear	0/10	0/10	33.11 ± 1.01	32.86 ± 1.88	with T2DM	Fenugreek, 5 g per serving were consumed 30 min before meals	placebo, 100 g yoghurt with flavors	NA	NA	8 weeks
Gholaman and Gholami, (2018)	87	45.5 ± 8.9	45.0 ± 7.7	27/43	27/44	30.5 ± 2.4	30.7 ± 3.2	with nonalcoholic fatty liver disease (NAFLD)	green cardamom, 3 g a day	placebo, 3 g a day	24 h food recall	aerobic physical activity at least 3 times a week for 30-45 minutes	3 months
Cheon et al., (2020)	149	43.7 (41.6-45.8)*	42.5 (40.0-44.9)*	0/76	0/73	32.4 (31.6-33.3)*	33.3 (32.3-34.3)*	Depends on BMI. None or more than one of metabolic risk factors	Euiiyin-tang, 3 g, three times daily	placebo, 3 g, three times daily	low-calorie diet during the study	NA	12 weeks

### 3.2 Evaluation of the risk of bias in the selected studies

**FIGURE 2:**
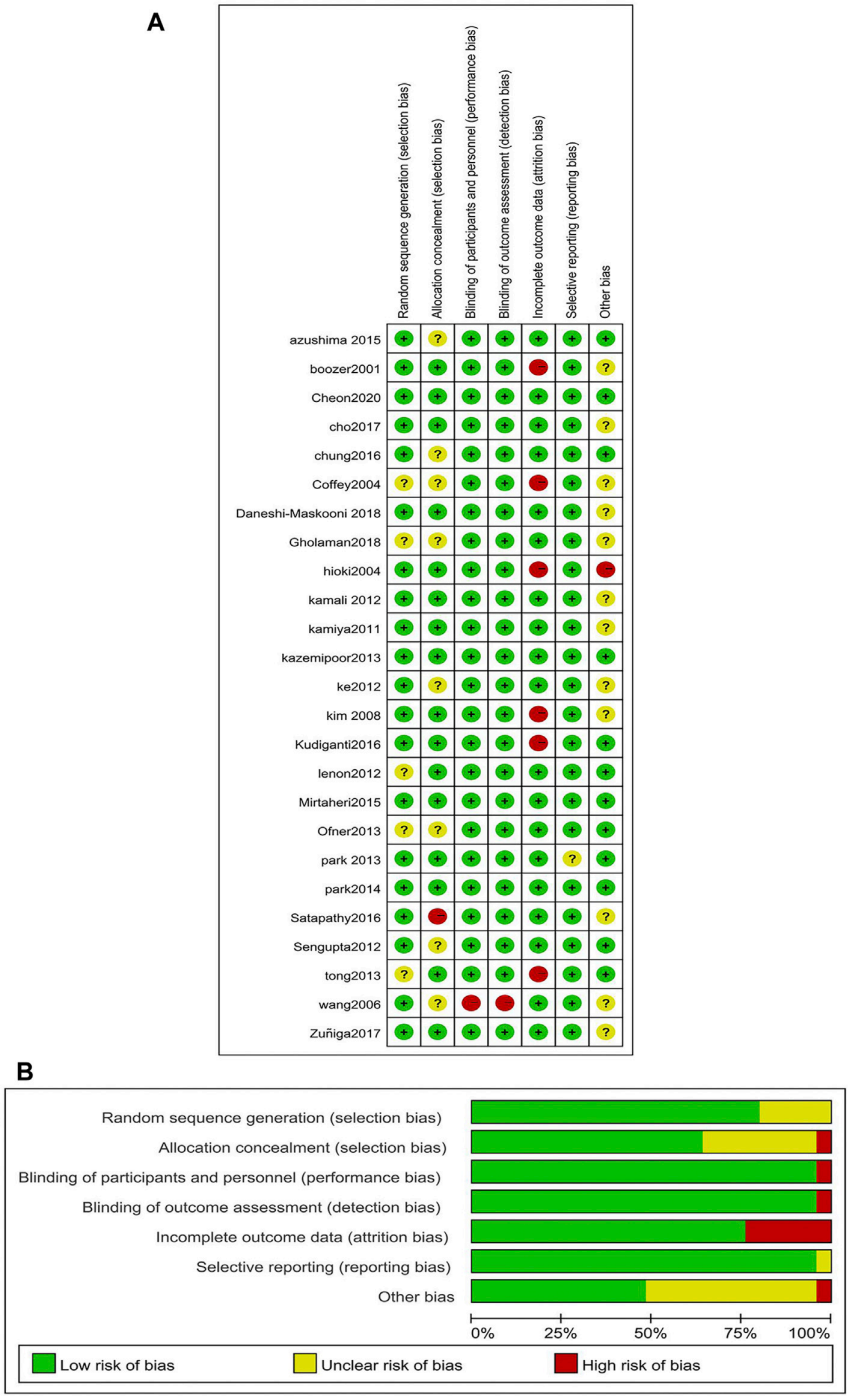
Risk of assessment for eligible studies. **(A)** Risk of bias summary; **(B)** Risk of bias graph.

We used the Cochrane Bias Risk Tool to assess the bias risk of 25 RCTs included. RCTs had a low overall risk of bias. Most RCTs are unclear about the risk of bias in sequence generation, allocation concealment, and reporting other biases because no detailed information is provided. However, six studies had a high risk of bias in the integrity of outcome data for participants, one study had a hidden high risk of bias in allocation concealment, one study had a high risk of bias in implementation and measurement, and the results of one study were evaluated by the blind method. One study had a high risk of bias in allocation concealment because it could not be conducted. In addition, most studies had a low risk of bias and incomplete outcome data. The risk of bias assessment is shown in Figure 2.

### 3.3 Meta-analysis results

#### 3.3.1 Main efficacy indicators—body mass index

**FIGURE 3:**
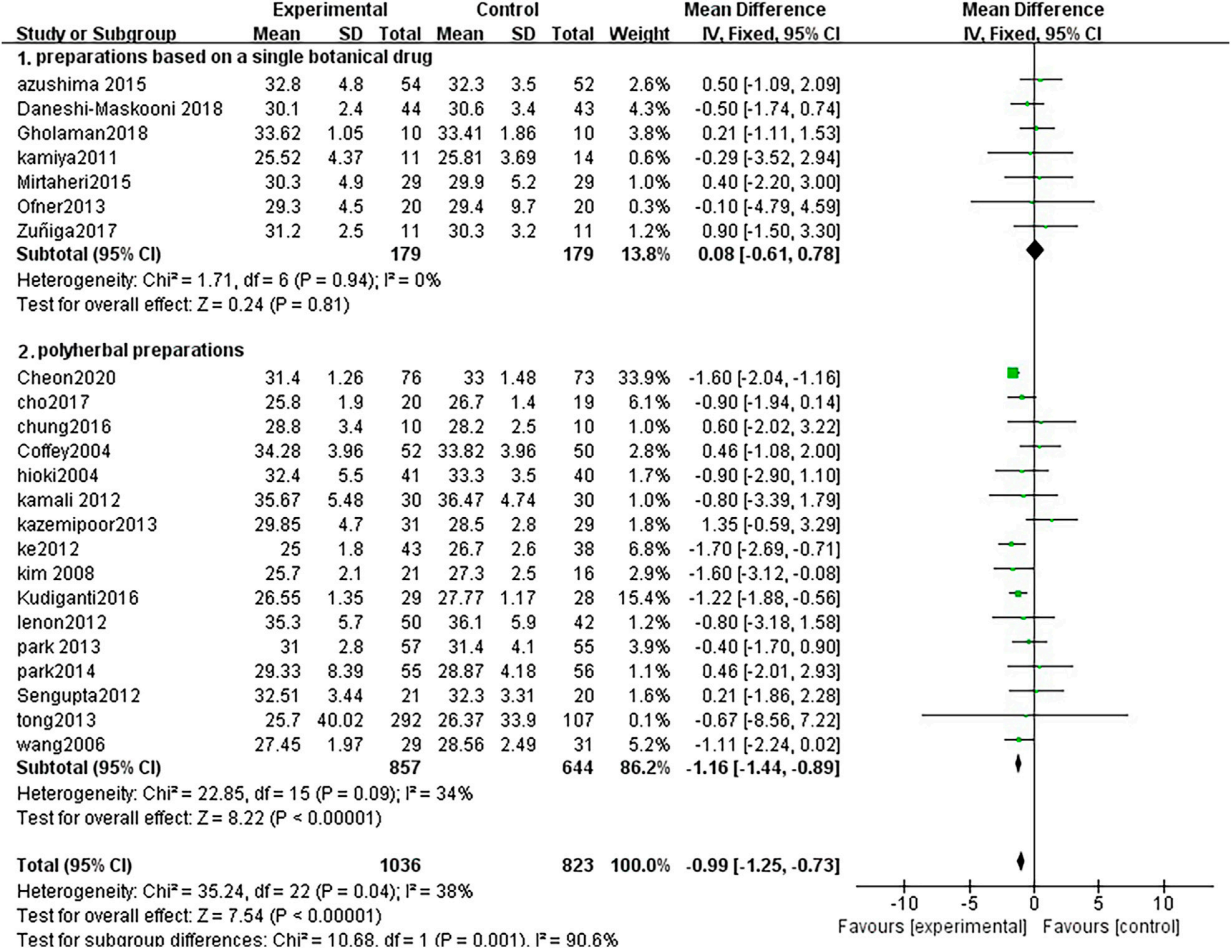
Forest plot of BMI. Comparison: TCM treatment (1.preparations based on a single botanical drug 2.polyherbal perparations). vs. placeo or blank control.

Twenty-three studies analyzed BMI index changes between TCM treatment (*n* = 1,036) and control (*n* = 823). The TCM groups include 7 preparations based on a single botanical drug groups and 16 polyherbal preparations groups. The decline of BMI in the polyherbal preparations groups was significantly higher than control groups (MD = −1.16, 95% CI = −1.44, −0.89; *p* < 0.00001; I^2^ = 34%). There was no significant difference in the preparations based on a single botanical drug groups (MD = 0.08, 95% CI = −0.61, 0.78; *p* = 0.81; I^2^ = 0%) (Figure 3)

#### 3.3.2 Secondary efficacy index

##### 3.3.2.1 Weight

**FIGURE 4:**
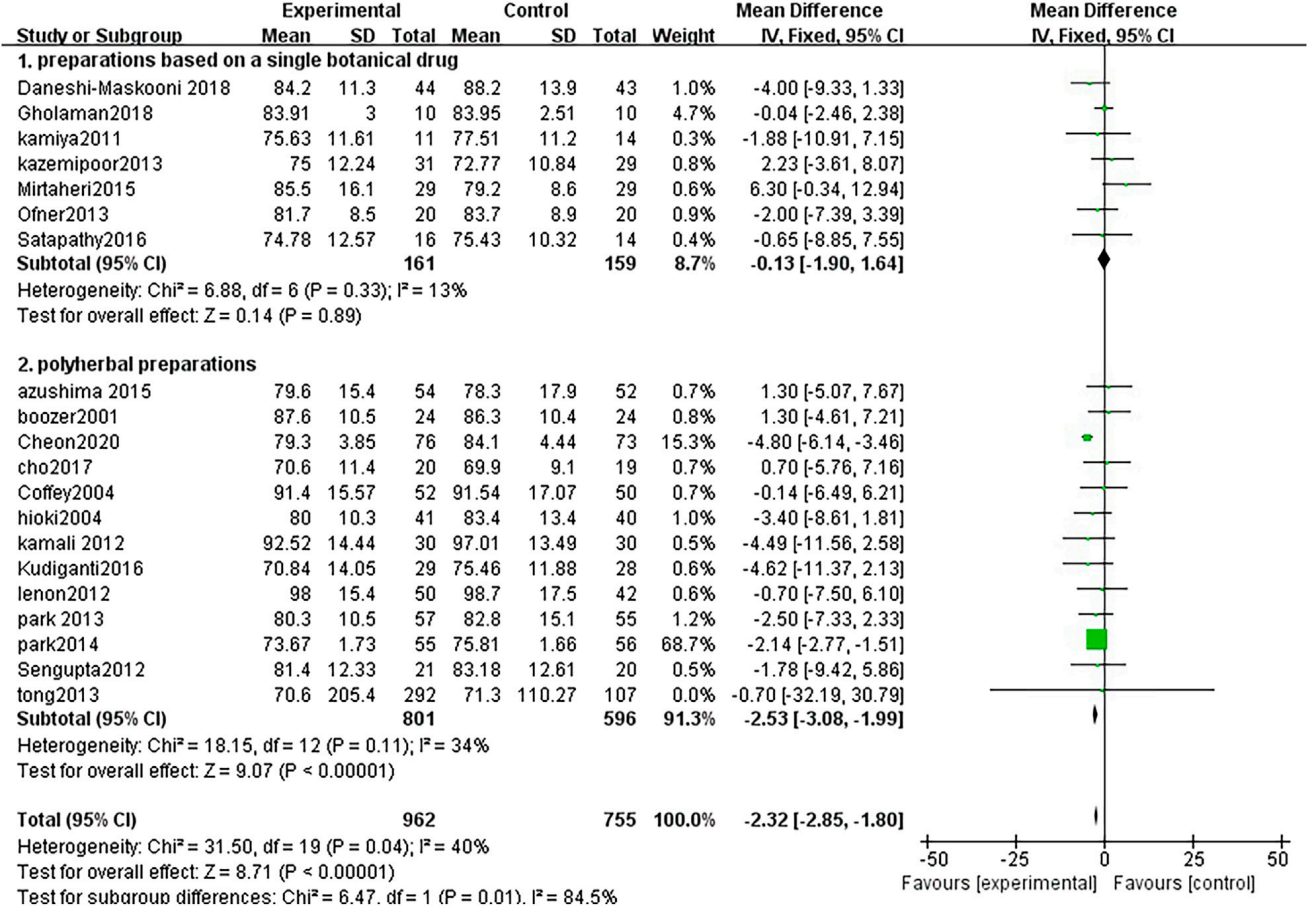
Forest plot of weight. Comparison: TCM treatment (1.preparations based on a single botanical drug 2.polyherbal perparations). vs. placeo or blank control.

Twenty studies analyzed weight changes between TCM treatment (*n* = 962) and control (*n* = 755). The TCM groups include 7 preparations based on a single botanical drug groups and 13 polyherbal preparations groups. The decline of weight in the polyherbal preparations groups was significantly higher than control groups (MD = −2.53, 95% CI = −3.08, −1.99; *p* < 0.00001; I^2^ = 34%). There was no significant difference in the preparations based on a single botanical drug groups (MD = −0.13, 95% CI = −1.90, 1.64; *p* = 0.89; I^2^ = 13%) (Figure 4)

##### 3.3.2.2 Waist circumference

**FIGURE 5:**
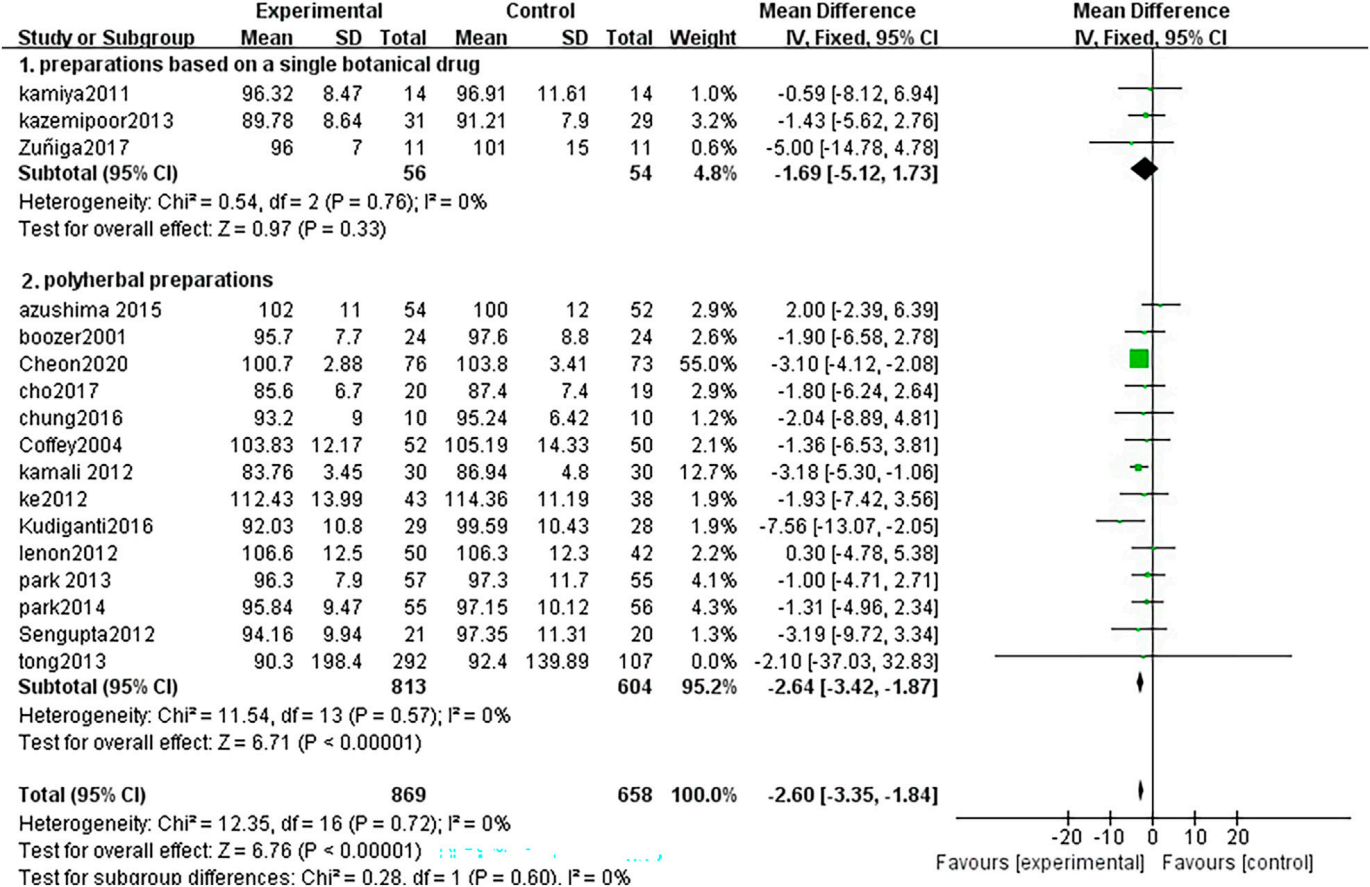
Forest plot of waist circumference. Comparison: TCM treatment (1.preparations based on a single botanical drug 2.polyherbal perparations). vs. placeo or blank control.

Seventeen studies analyzed waist circumference changes between TCM treatment (*n* = 869) and control (*n* = 658). The TCM groups include 7 preparations based on a single botanical drug groups and 13 polyherbal preparations groups. The decline of Weight in the polyherbal preparations groups was significantly higher than control groups (MD = −2.64, 95% CI = −3.42, −1.87; *p* < 0.00001; I^2^ = 0%). There was no significant difference in the preparations based on a single botanical drug groups (MD = −1.69, 95% CI = −5.12, 1.73; *p* = 0.33; I^2^ = 0%) (Figure 5)

##### 3.3.2.3 Hip circumference

**FIGURE 6:**
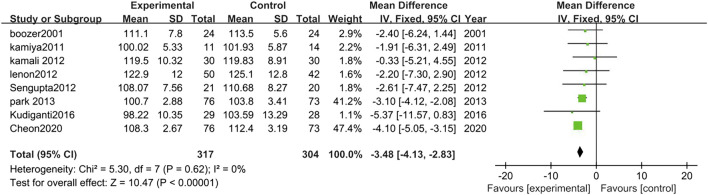
Forest plot of hip circumference. Comparison: TCM treatment vs. placeo or blank control.

Eight studies analyzed the changes in Hip circumference between TCM treatment (*n* = 317) and control (*n* = 304). The hip circumference reduction in the TCM group was significantly more than control groups (MD = −3.48, 95% CI = −4.13, −2.83; *p* < 0.00001; I^2^ = 0%) (Figure 6).

##### 3.3.2.4 Body fat rate

**FIGURE 7:**
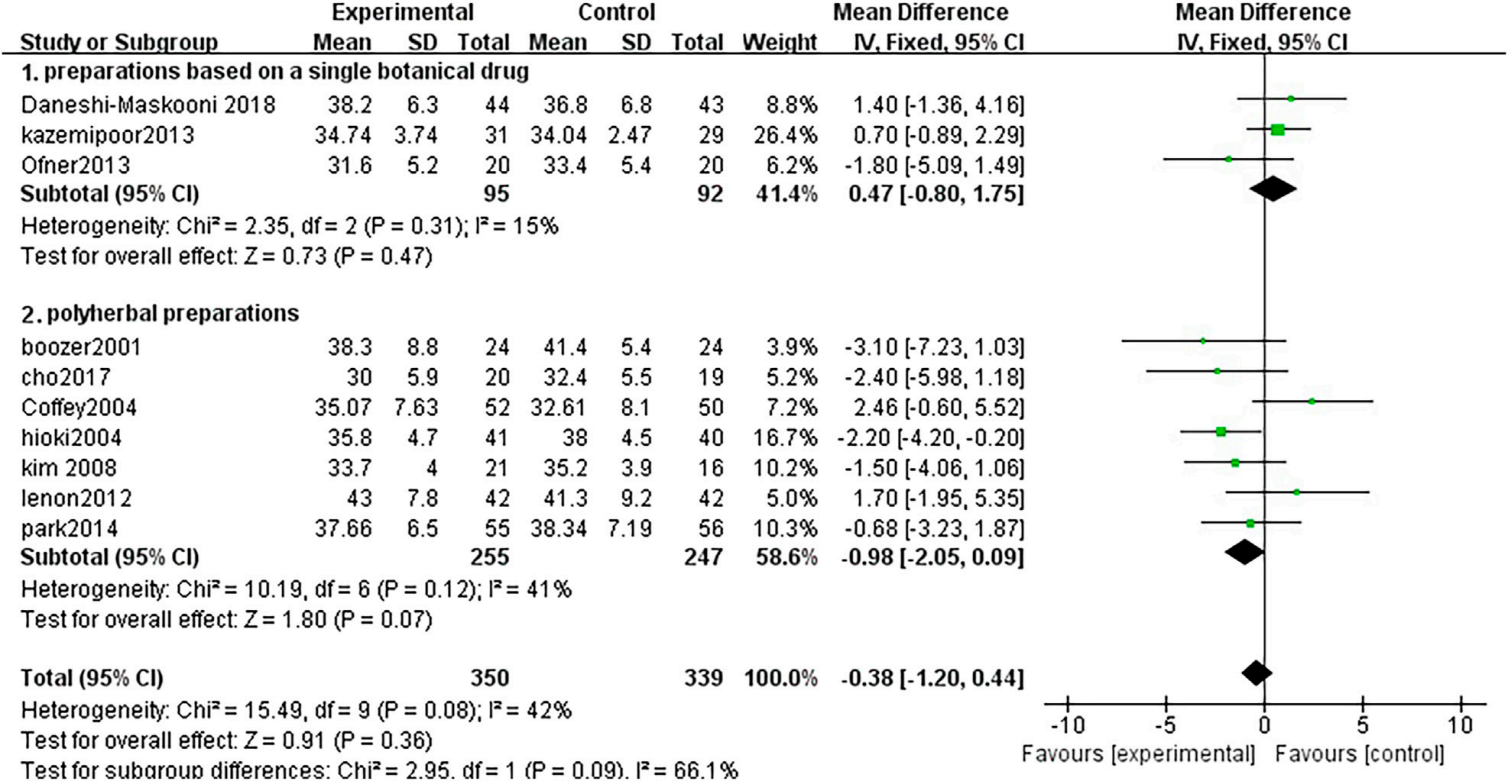
Forest plot of body fat rate. Comparison: TCM treatment (I.preparations based on a single botanical drug 2.polyherbal perparations). vs. placeo or blank control.

Ten studies analyzed the changes in the body fat rate between TCM treatment (*n* = 50) and control (*n* = 339). The TCM groups include 3 preparations based on a single botanical drug groups and 7 polyherbal preparations groups. There was no significant difference in the polyherbal preparations groups (MD = 0.47, 95% CI = −0.80, 1.75; *p* = 0.47; I^2^ = 15%). There was no significant difference in the preparations based on a single botanical drug groups (MD = −0.98, 95% CI = −2.05, 0.09; *p* = 0.07; I^2^ = 41%) (Figure 7)

##### 3.3.2.5 Triglycerides

**FIGURE 8:**
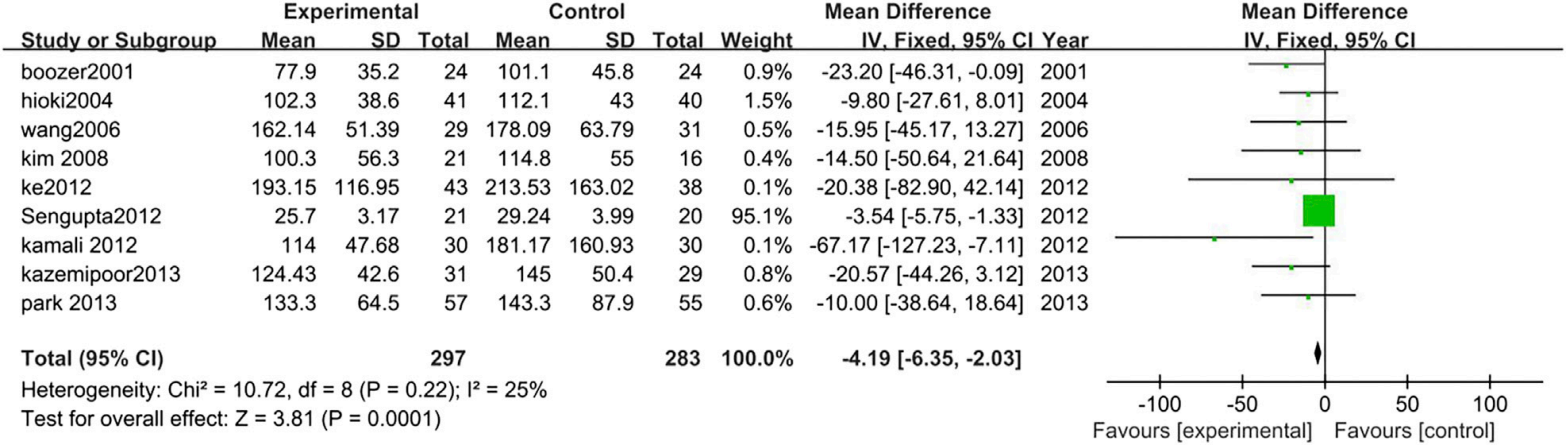
Forest plot of TG. Comparison: TCM treatment vs. placeo or blank control.

Nine studies analyzed the changes in TG levels between TCM treatment (*n* = 297) and control (*n* = 283). The TCM treatment groups were polyherbal preparations. The decrease of TG in the polyherbal preparations groups was significantly higher than control groups (MD = −4.19, 95% CI = −6.35, −2.03; *p* = 0.0001; I^2^ = 25%) (Figure 8).

##### 3.3.2.6 Total cholesterol

**FIGURE 9:**
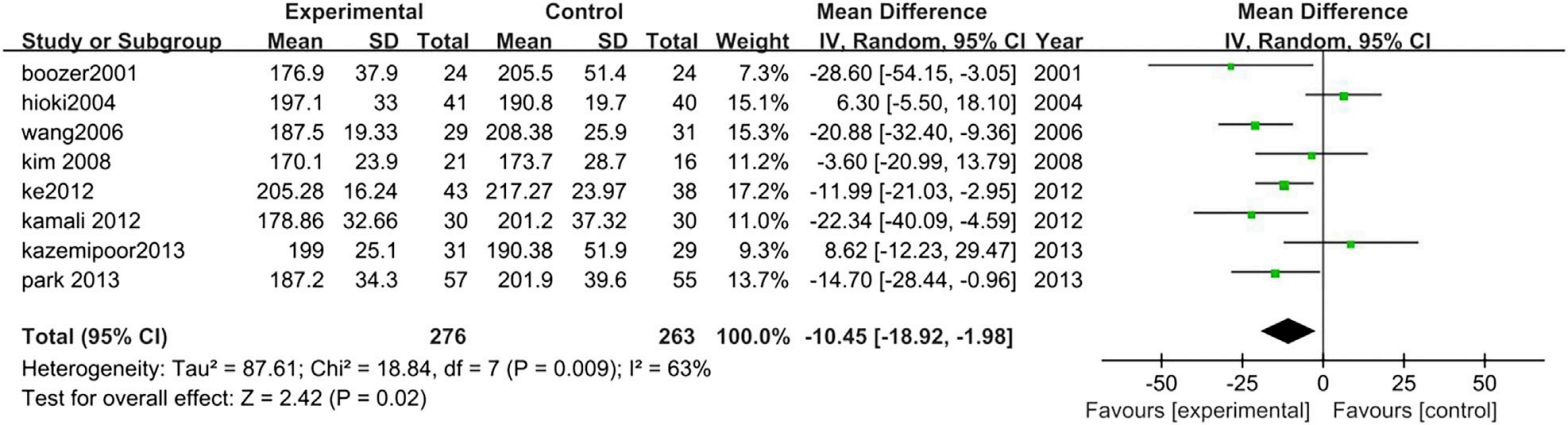
Forest plot of TCHO. Comparison: TCM treatment vs. placeo or blank control.

Eight studies analyzed the changes in TCHO levels between TCM treatment (*n* = 276) and control (*n* = 263). The TCM treatment groups were all polyherbal preparations. The decrease of TCHO in the TCM intervention group was significantly higher than control group (MD = −10.45, 95% CI = −18.92, −1.98; *p* = 0.02; I^2^ = 63%) (Figure 9).

##### 3.3.2.6 Low-density lipoprotein

**FIGURE 10:**
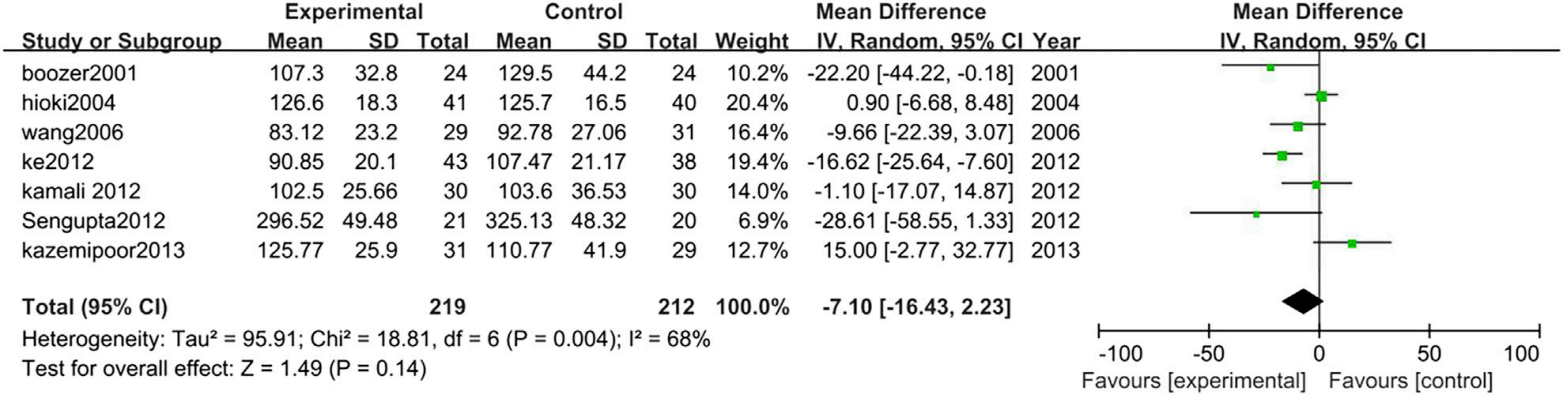
Forest plot of LDL. Comparison: TCM treatment vs. placeo or blank control.

Seven studies analyzed the changes in LDL between TCM treatment (*n* = 219) and control (*n* = 212). The TCM treatment groups were all polyherbal preparations. There was no significant difference in LDL between TCM intervention group and control group (MD = −7.10, 95% CI = −16.43, 2.23; *p* = 0.14; I^2^ = 68%) (Figure 10).

##### 3.3.2.7 High-density lipoprotein

**FIGURE 11:**
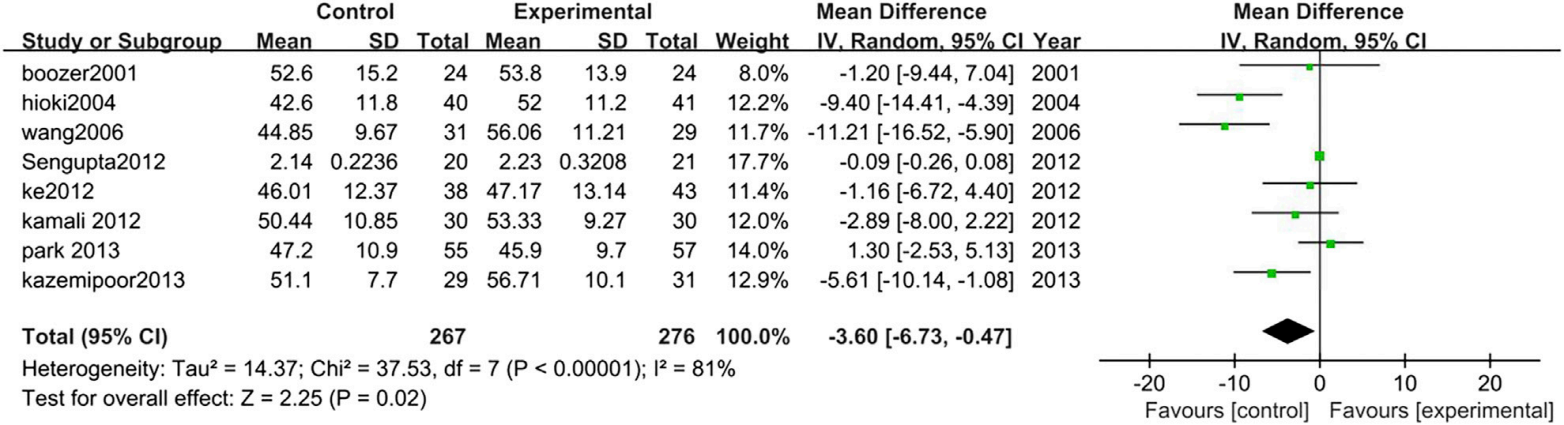
Forest plot of HDL. Comparison: TCM treatment vs. placeo or blank control.

Eight studies analyzed HDL changes between TCM treatment (*n* = 276) and control (*n* = 267). The TCM treatment groups were all polyherbal preparations. The decrease of HDL in the TCM group was significantly higher than control group (MD = −3.60, 95% CI = −6.73, −0.47; *p* = 0.02; I^2^ = 81%) (Figure 11).

##### 3.3.2.8 Fasting blood glucose

**FIGURE 12:**
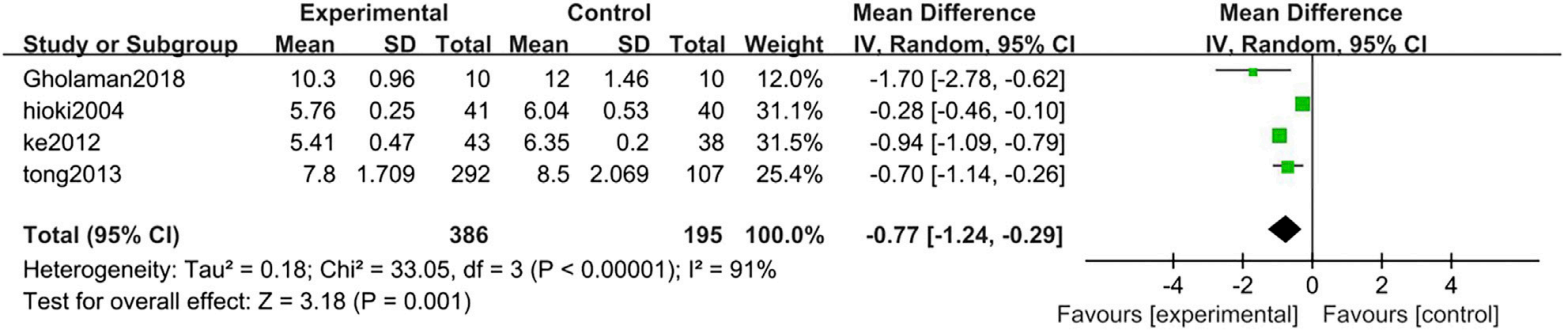
Forest plot of FBG. Comparison: TCM treatment vs. placeo or blank control.

Four studies analyzed FBG levels in TCM treatment group (*n* = 386) compared with control group (*n* = 195). The FBG level in the TCM group was lower than control group (MD = −0.77, 95% CI = −1.24, −0.29; *p* = 0.001; I^2^ = 91%) (Figure 12).

##### 3.3.2.9 Glycated hemoglobin

**FIGURE 13:**
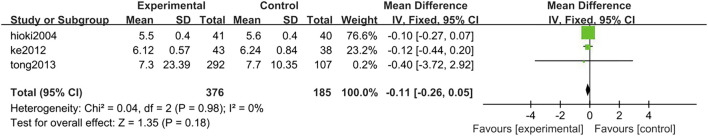
Forest plot of HbAlc. Comparison: TCM treatment vs. placeo or blank control.

Three studies analyzed HbA1c levels in the TCM treatment group (*n* = 376) compared with the control group (*n* = 185). There was no significant difference between TCM group and control group (MD = −0.04, 95% CI = −0.21, 0.14; *p* = 0.69; I^2^ = 0%) (Figure 13).

##### 3.3.2.10 Blood pressure

**FIGURE 14:**
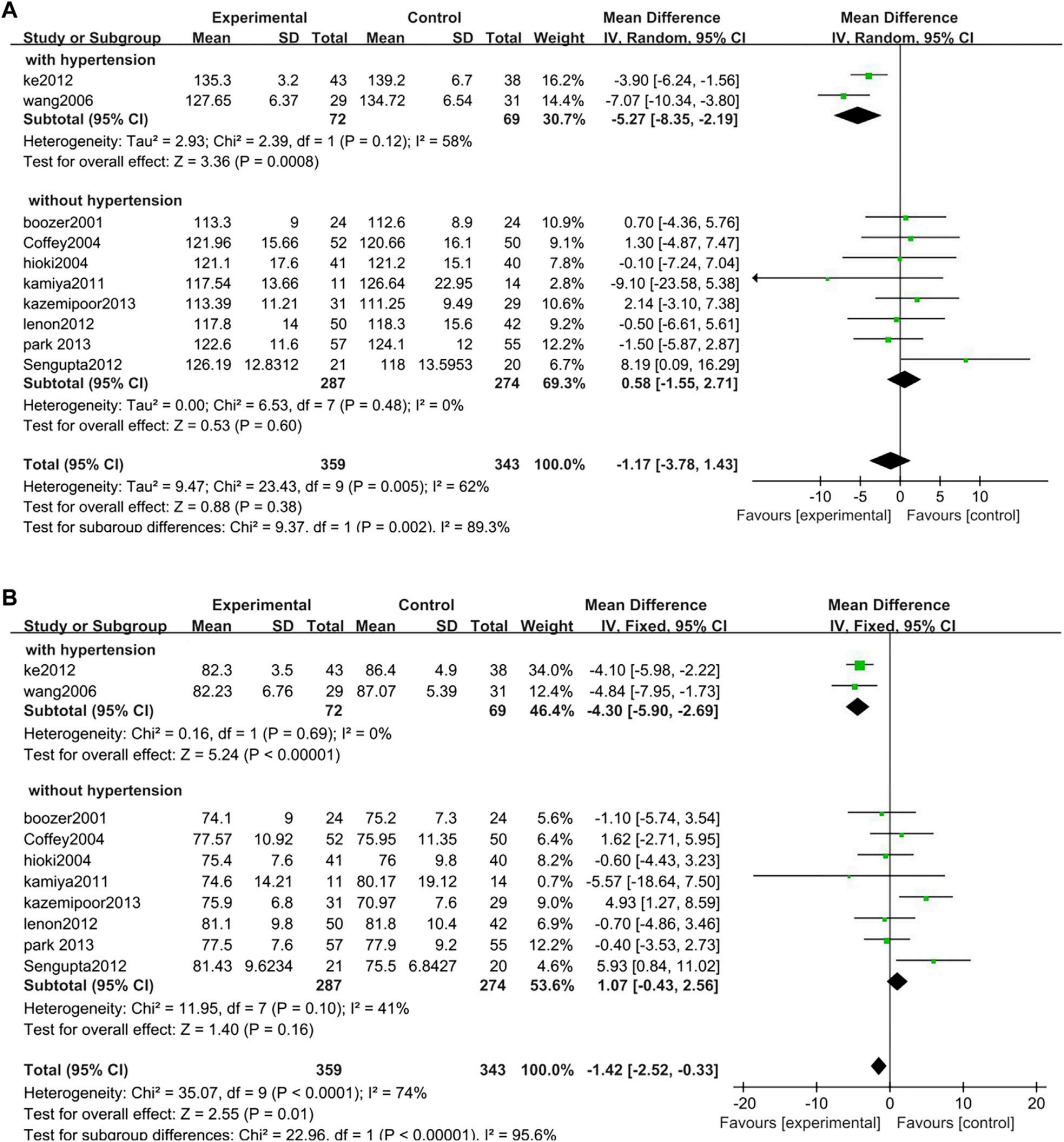
Forest plot of blood pressure. Comparison: TCM treatment vs. placeo or blank control. **(A)** Forest plot of SBP. **(B)** Forest plot of DBP.

Ten studies analyzed blood pressure in overweight and obese people with TCM treatment (*n* = 359) and control (*n* = 343), of which two studies were focusing on overweight and obesity combined hypertension and the remaining eight studies were on overweight and obesity with normal blood pressure. The results of two study populations with hypertension are as follows: SBP: MD = −5.27, 95% CI = −8.35, −2.19; *p* = 0.0008; I^2^ = 58%, DBP: MD = −4.30, 95% CI = −5.90, −2.69; *p* < 0.00001; I^2^ = 0%. The results of the other eight studies: SBP: MD = −5.27, 95% CI = −8.35, −2.19; *p* = 0.0008; I^2^ = 58%, DBP: MD = −4.30, 95% CI = −5.90, −2.69; *p* < 0.00001; I^2^ = 0%. According to the results of the current analysis, there was no significant difference in blood pressure before and after TCM intervention in overweight and obese people with normal blood pressure. For overweight and obese people with hypertension, TCM has a certain antihypertensive effect (Figure 14).

##### 3.3.2.11 Safety

**FIGURE 15:**
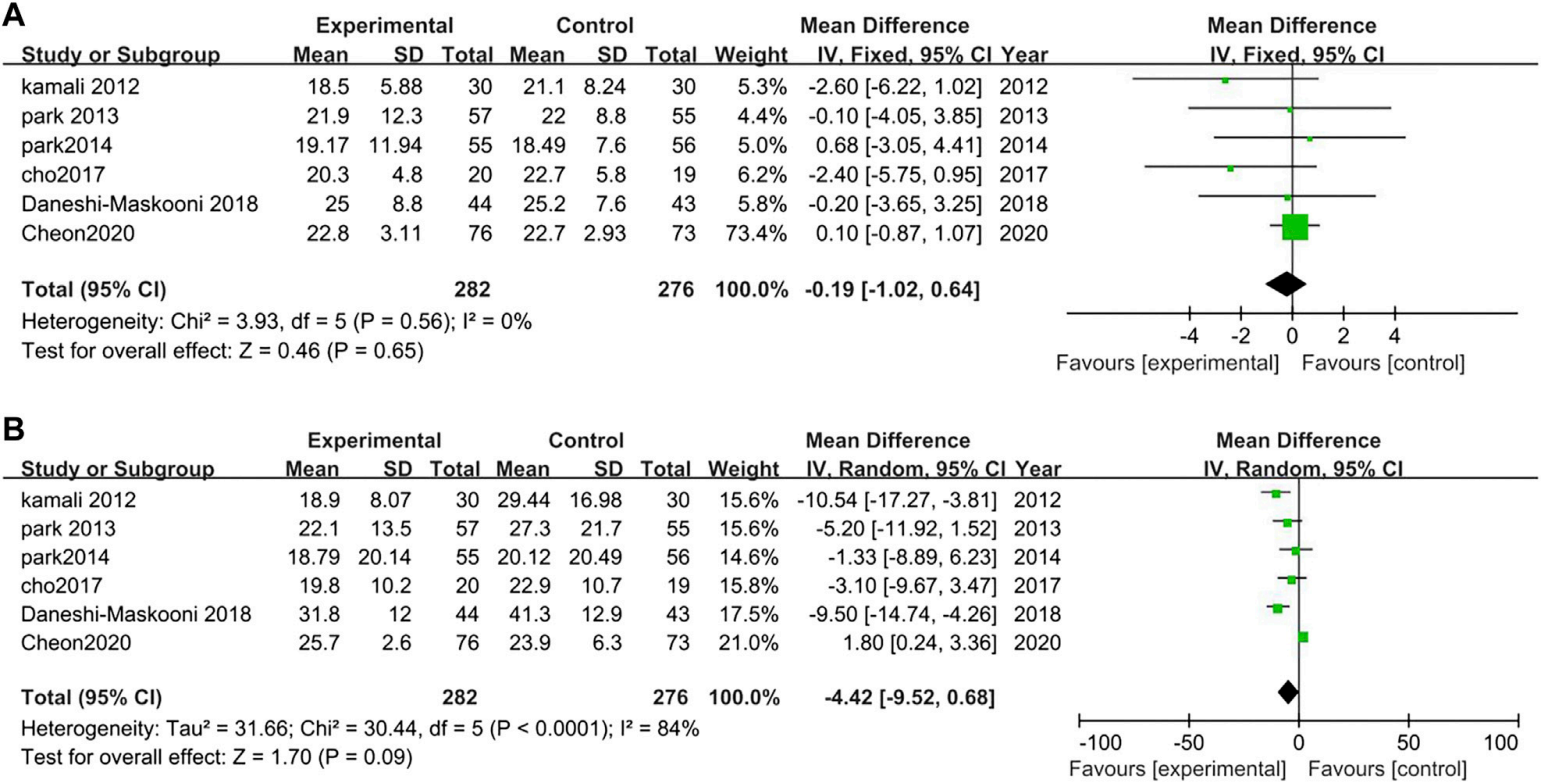
Forest plot for safety. Comparison: TCM treatment vs. placeo or blank control.**(A)** Forest plot of AST. **(B)** Forest plot of ALT.

Six studies analyzed the safety between TCM treatment (*n* = 282) and control (*n* = 276).The results suggested that there was no difference of aspartate aminotransferase (AST) and alanine aminotransferase (ALT) before and after the TCM treatment (AST: MD = −0.19, 95% CI = −1.02, 0.64; *p* = 0.65; I^2^ = 0%, ALT: MD = −4.42, 95% CI = −9.52, 0.68; *p* = 0.09; I^2^ = 84%). However, in the study of Cheon et al. (2020) one patient presented with elevated ALT and AST after TCM treatment. Tong et al. (2013) reported that two of these patients had transient ALT and AST elevation (Figure 15).

##### 3.3.2.12 Adverse effects

Adverse effects were reported in 19 of the 25 studies. Mirtaheri et al. (2015) reported no adverse effects, other five studies (Wang et al., 2006, Kamiya et al., 2011, Ofner et al., 2013; Satapathy et al., 2016; Gholaman and Gholami, 2018) did not mention the occurrence of adverse effects. Most of the reported adverse reactions were mild, mainly in the digestive system, such as dry mouth, epigastric pain, nausea and indigestion, constipation, or diarrhea. As well as neurological symptoms such as dizziness or headache, insomnia, etc., and mood changes. See Table 2 for details.

**TABLE 2:** Adverse reaction of included study.

Study ID	TCM treatment	Control
Coffey et al., (2004)	A total of 1 96 adverse events observed over the course of this study. Of the 1.02 patients in the study, 78 (76%) suffered at least one AE and 30 (29%) suffered at least one PTRAE over the course of the study. There was no difference in the occurrence of any adverse event between the two groups (77% for active vs. 76% for control, PY 0.91). There was no difference in the occurrence of any PTRAE between the two groups (33% for active vs. 26% for control, PY 0.46). Of the 78 subjects who experienced adverse events, 56 had multiple adverse events.	
	One subject had two adverse events classified as serious: 'Low Back Pain' and 'Compression Fracture of LI '	One subject had three adverse events. 'Exacerbated Depression', 'Atrial Fibrillation', and 'Exacerba- tion of Asthma'
Hioki et al., (2004)	loose bowels (*n* = 3)	No serious adverse effects
Kim et al, (2008)	Palpitation (*n* = 1), Headache (*n* = 7), Dull head (*n* = 4), I nsomnia (*n* = 4), Dizziness (*n* = 4), Nervousness (*n* = 1), Nausea (*n* = 2), Vomiting (*n* = 2), Anorexia (*n* = 1), Constipat ion (*n* = 12), Dry mouth (*n* = 6)	Insomnia (*n* = 2)
		Nervousness (*n* = 1)
		Constipation (*n* = 3)
		Eruption (*n* = 1)
		Dry mouth (*n* = 1)
Kamali et al, (2012)	Without statistically significant differences in rates of any adverse events.	
Ke et al, (2012)	There were five patients that felt fatigue, hunger and dizziness, and which was recovered after giving normal diets. Besides, no serious side effect was found.	No serious adverse effects
Sengupta et al, (2012)	There were no major adverse events reported. Some minor adverse events such as gastric irritation, abdominal pain and back pain were reported by few subjects. These minor events were distributed evenly between the placebo and treatment groups.	
Lenon 2012	nausea (*n* = 4)	decreasein appetite (*n* = 2)
	headache (*n* = 9)	
Tong et al, (2013)	There were no medium or serious adverse events reported. Twenty-four mild adverse events (6.69%) were reported in experimental group versus 7 mild adverse events (5.83%) reported (*p* = 0.743). In the Experimental group, there were two cases with transient slight ALT elevation and two with AST elevation.	
Kazemipoor et al, (2013)	only the placebo participants experienced skin allergy to the placebo product , and no important adverse events were reported during the physical examinations.	
Park et al, (2013)	no adverse effects were reported.	
Park et al, (2014)	epigastric pain (*n* = 7)	dyspepsia and epigastric pain (*n* = 3)
	headache (*n* = 2)	and headache (*n* = 1)
	diarrhea (*n* = 3)	
	nausea and vomiting (*n* = 2),	
	palpitations (*n* = 1)	
Azushima et al, (2015)	3 patients in the experienced minor adverse events (gastric irritation, constipation, and elevation of serum hepatic enzyme level).	no adverse events reported in the control group
Kudiganti et al, (2016)	7 minor adverse events occurred in 5 people:	9 adverse events occurred in 6 people : Dyspepsia (*n* = 2), Nausea (*n* = 1), Gastritis (*n* = 1), Pain Headache (*n* = 1), Itching (*n* = 1), Rash on forearm (*n* = 1), Giddiness (*n* = 1), Feet swelling (*n* = 1)
	Acidity (*n* = 2)	
	Dyspepsia (*n* = 3)	
	Nausea (*n* = l)	
	Gastritis (*n* = 1)	
Chung et al, (2016)	There were no adverse sign except burning sensation, indigestion and fatigue for severa l volunteer, and no significantly different between two groups.	
Cho et al, (2017)	gastrointestinal symptoms, such as dyspepsia, nausea, epigastricsoreness ,diarrhea ,and constipation (*n*=4)	gastrointestina l symptoms,
	upper respiratory tract infections (*n* = 3)	such as dyspepsia, nausea,
	headache and dizziness (*n* = 2)	epigastricsoreness, diarrhea, and constipation (*n* = 8)
	skin rash (*n* = 2)	upper respiratory tract infections (*n* = 4)
	musculo skeletal pain (*n* = 1)	headache and dizziness (*n* = 2)
		musculo skeletal pain (*n* =1)
		fatigue (*n* = 1)
Zuniga et al, (2017)	two subjects (16.7"/o) reported diarrhea, pyrosis, and polydipsia, and one subject (8.3%) reported abdominal distension and headache	Two subjects (16.7%) from the placebo group reported headache and one patient (8.3%) reported diarrhea and pyrosis.
Daneshi-Maskooni et al, (2018)	No side effects associated with the treatment.	Only one patient reported nausea and constipation in one of his followed up
Cheon et al, (2020)	In total, 10 (6.7"/o) participants experienced adverse events during the trial, which included 4 (5.3%) from the	
	Headache (*n* = I)	Diarrhea (*n* = 1)
	Diarrhea (*n* = J)	Aspartate aminotransferase increased (*n* = 1)
	Herpes zoster (*n* = 1)	Alanine aminotransferase increased (*n* =1)
	Cholelithiasis (*n* = 1)	Concussion (*n* = 1)
	Dermatitis allergic (*n* = 1)	Peripheral swelling (*n* =1)
		Hypertonic bladder (*n* = 1)
		Uterine leiomyoma (*n* = 1)
		Uterine polyp (*n* = 1)

##### 3.3.2.13 Publication bias

**FIGURE 16:**
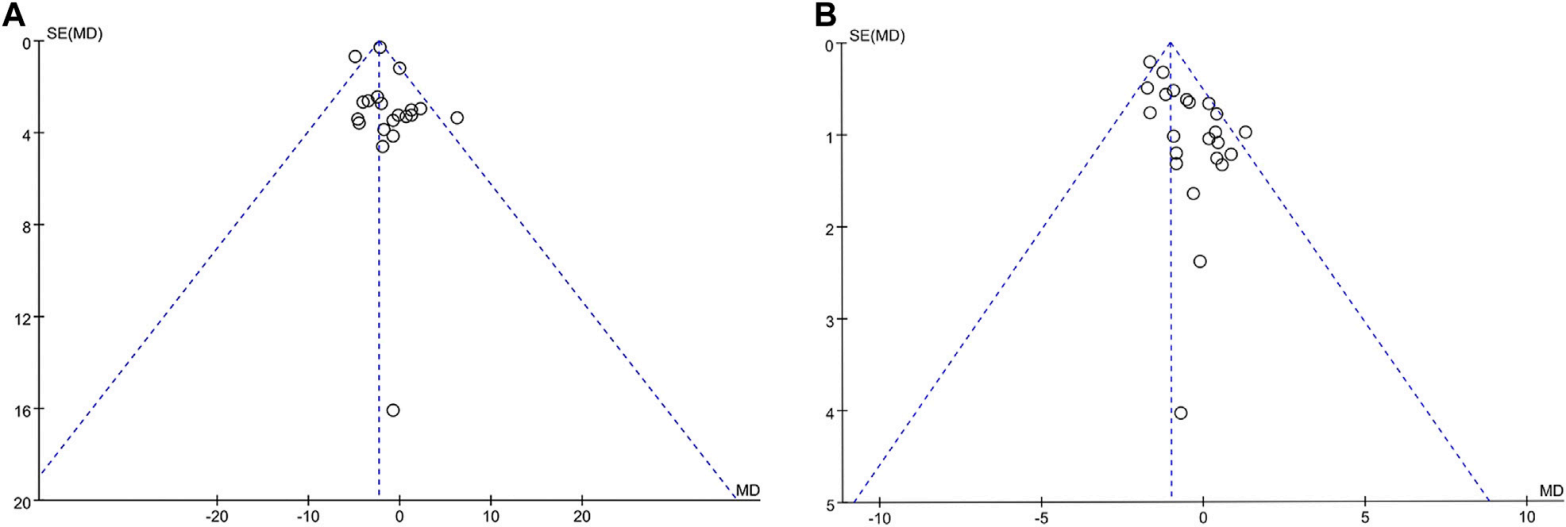
Funel plot of publication bias. Comparison: TCM treatment placeo or blank control. **(A)** Funel plot in publication bias in weight. **(B)** Funel plot on publication bias in BMI.

The software was used to analyze the publication bias of 20 studies on the main outcome - BMI and 23 studies on the outcome—weight (Figure 16).

##### 3.3.2.14 GRADE of the outcomes

We used GRADE Profiler 3.6 to evaluate all outcome indicators in the following respects: 1) downgrading the quality of evidence, risk of bias, inconsistencies, indirectness, inaccuracy, and publication bias. 2) upgrading the quality of evidence, large effect, possible confounding change effect, and dose-response gradient. After a comprehensive analysis, the evidentiary body was formed and found that all outcome indicators had low quality or extremely low quality. See Table 3 for details.

**TABLE 3:** Grade of the outcomes.

Quality assessment							No of patients		Effect		Quality Importance
No of studies	Design	Risk of bias	Inconsistency	Indirectness	Imprecision	Other considerations	Experimental	Contral	Relative (95% CI)	Absolute	
weight (Better indicated by lower values)											
20	randomised trials	very serious	no serious inconsistency	no serious indirectness	no serious imprecision	none	962	755	–	MD 2.32 lower (2.85 to 1.8 lower)	⊕⊕○○ LOW
BMI (Better indicated by lower values)											
23	randomised trials	very serious^1^	no serious inconsistency	no serious indirectness	no serious imprecision	none	1036	823	–	MD 1 lower (1.26 to 0.74 lower)	⊕⊕○○ LOW
Waist circumference (Better indicated by lower values)											
17	randomised trials	very serious^1^	no serious inconsistency	no serious indirectness	no serious imprecision	none	869	658	–	MD 2.62 lower (3.36 to 1.88 lower)	⊕⊕○○ LOW
Hip circumference (Better indicated by lower values)											
8	randomised trials	very serious^1^	no serious inconsistency	no serious indirectness	very serious^2^	none	317	304	–	MD 3.48 lower (4.13 to 2.83 lower)	⊕⊕○○ VERY LOW
waist to hip ratio (Better indicated by lower values)											
7	randomised trials	very serious^1^	no serious inconsistency	no serious indirectness	very serious^2^	none	266	245	–	MD 0.01 higher (0 to 0.01 higher)	⊕⊕○○ VERY LOW
body fat (Better indicated by lower values)											
10	randomised trials	very serious^1^	no serious inconsistency	no serious indirectness	very serious^2^	none	350	339	–	MD 0.38 lower (1.2 lower to 0.44 higher)	⊕⊕○○ VERY LOW
Total cholesterol (Better indicated by lower values)											
8	randomised trials	very serious ^1^	serious^3^	no serious indirectness	very serious^2^	none	276	263	–	MD 10.45 lower (18.92 to 1.98 lower)	⊕⊕○○ VERY LOW
Triglycerides (Better indicated by lower values)											
9	randomised trials	very serious^1^	no serious inconsistency	no serious indirectness	very serious^2^	none	297	283	–	MD 4.19 lower (6.35 to 2.03 lower)	⊕⊕○○ VERY LOW
high-density lipoprotrein (Better indicated by lower values)											
8	randomised trials	very serious^1^	very serious^4^	no serious indirectness	very serious^2^	none	276	267	–	MD 3.6 higher (0.47 to 6.73 higher)	⊕⊕○○ VERY LOW
low-density lipoprotein (Better indicated by lower values)											
7	randomised trials	very serious^1^	serious^3^	no serious indirectness	very serious^2^	none	219	212	–	MD 7.1 lower (16.43 lower to 2.23 higher)	⊕⊕○○ VERY LOW
systolic blood pressure (Better indicated by lower values)											
10	randomised trials	very serious^1^	serious^3^	no serious indirectness	very serious^2^	none	359	343	–	MD 3.15 lower (4.56 to 1.74 lower)	⊕⊕○○ VERY LOW
diastolic blood pressure (Better indicated by lower values)											
10	randomised trials	very serious^1^	no serious inconsistency	no serious indirectness	very serious^2^	none	359	343	–	MD 1.42 lower (2.52 to 0.33 lower)	⊕⊕○○ VERY LOW
Alanine aminotransferase (Better indicated by lower values)											
6	randomised trials	very serious^1^	no serious inconsistency	no serious indirectness	very serious^2^	none	282	276	–	MD 0.19 lower (1.02 lower to 0.64 higher)	⊕⊕○○ VERY LOW
Aspartate aminotransferase (Better indicated by lower values)											
6	randomised trials	very serious^1^	very serious^4^	no serious indirectness	very serious^2^	none	282	276	–	MD 4.42 lower (9.52 lower to 0.68 higher)	⊕⊕○○ VERY LOW
dropout rate											
24	randomised trials	very serious^1^	no serious inconsistency	no serious indirectness	no serious imprecision	none	201/1186 (16.9%)	156/979 (15.9%)	RR 1.06 (0.88 to 1.29)	10 more per 1000 (from 19 fewer to 46 more)	⊕⊕○○ LOW
								10.1%	6 more per 1000 (from 12 fewer to 29 more)

1 most articles are biased

2 sample size is small

3 Heterogeneity is large

4 Heterogeneity is very large

##### 3.3.2.15 Sensitivity analysis

**FIGURE 17:**
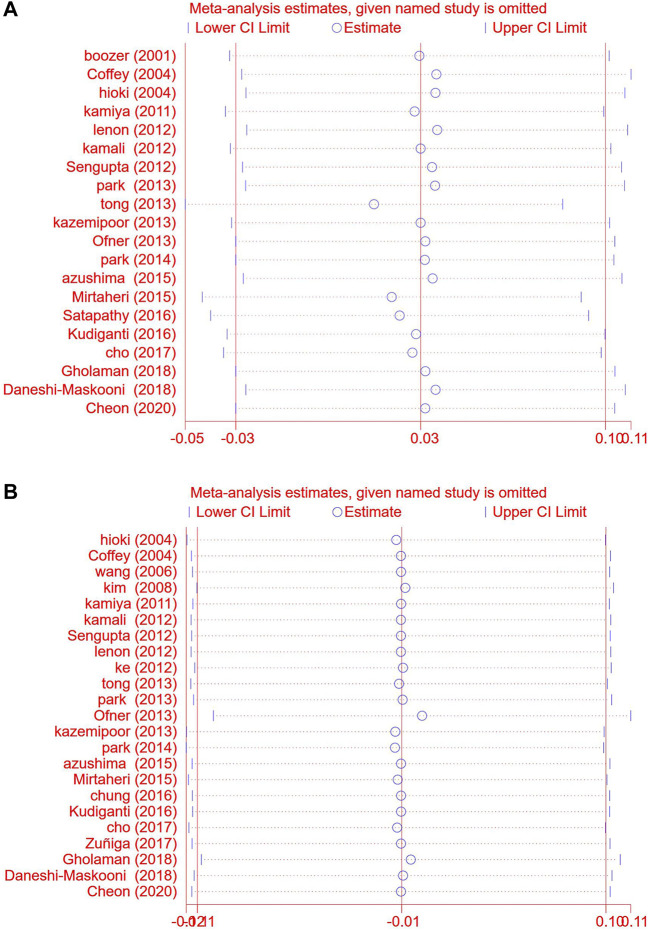
.Sensitivity analysis chart. Comparison: TCM treatment placeo or blank control. **(A)** Sensitivity analysis chart of weight. **(B)** Sensitivity analysis chart of BMI.

We used stata15 SE software to conduct sensitivity analysis on its main outcome-weight and BMI (Figure 17).

## 4 Discussion

### 4.1 Research results

We conducted a systematic evaluation based on 25 current RCTs including 1,947 subjects. We compared the efficacy of weight reduction and the effects on blood glucose, blood lipids, and blood pressure in overweight and obese patients in the TCM group and control group. TCM treatment is divided into preparations based on a single botanical drug and polyherbal preparations. From the analysis results, TCM preparations for overweight and obese people can effectively reduce weight, BMI, waist circumference, and hip circumference after certain periods. There was no significant difference in the preparations based on a single botanical drug group compared to the control group. At the same time, the obesity-related risk factors were analyzed. Compared with control, TCM preparations can reduce FBG and blood pressure, and regulate lipid metabolism disorder in overweight and obese patients with diabetes mellitus. There is no significant difference in liver function before and after the intervention of TCM, which has high safety and fewer adverse reactions.

### 4.2 Analysis of the curative effect of TCM

#### 4.2.1 Lose weight

In recent years, the BMI of the global population has been increasing (Jaacks et al., 2019). Compared with western countries, the obesity rate in China is low, but the growth trend is obvious (Wang et al., 2021). From 1989 to 2015, the number of overweight adults increased from 16.8% to 31%, and the number of obese adults increased from 3.8% to 11.3% (Pan et al., 2021). Therefore, it is urgent to explore new therapies that can effectively improve the treatment of obesity.

The systematic evaluation proved that 23 studies analyzed the BMI of 1,036 people in the TCM group and 823 people in the control group. The BMI of the TCM group decreased significantly more than the control group. And the decline was more pronounced in the polyherbal preparations group 20 of the 25 included studies analyzed and compared the weight of 962 people in the TCM treatment group and 755 people in the control group, it was found that the weight loss in the TCM group was significantly more than that in the control group. Likewise, the decline was more pronounced in the polyherbal preparations group. 17 studies analyzed the waist circumference of 869 people in the TCM group and 658 people in the control group. The waist circumference of the TCM group decreased significantly more than the control group. There was also a significant change in hip circumference in the herbal treatment group compared to the control group. However, there is some heterogeneity in the combination of these studies. These studies were from different countries and geographic regions, and the different BMI levels and comorbidity differences in overweight and obese people may be the source of these heterogeneities.

Interestingly, the study found that the overall herbal treatment was superior to the control group, but it appears the herbal compound has a larger variety of drugs and a more complex composition, which may have stronger effects. In addition, herbal compounding will also play a certain synergistic effect on each other compared to single prescriptions, which will form a complex pharmacological network. A single herb may not achieve this effect. In addition, TCM treatment is concerned with the relationship between the combination of ingredients, and an appropriate combination also improves the efficacy to some extent. This complex effect may be the key to impacting the synergistic effect of TCM multi-targets. In theory, choosing and creating a single target additive effect could realize the scientific compatibility of TCM and improve the curative effect and attenuate the toxicity (Weng et al., 2018).

At present, the main methods to treat obesity include lifestyle management, weight-loss drugs, bariatric surgery, reducing food intake and absorption, and improving its utilization (Bray et al., 2018). Weight-loss drugs act through peripheral and central mechanisms. They may achieve different degrees of rapid weight loss by increasing satiety, energy consumption, action pathway, and inhibiting calorie absorption (Heffron et al., 2020). However, they also have the characteristics of large side effects and many contraindications. Weight-loss drugs are suitable for a limited population and can potentially increase the risk of some diseases.

Orlistat may reduce body weight by inhibiting dietary fat absorption (Lavie et al., 2015), increasing adiponectin levels, reducing inflammation, and improving insulin sensitivity (Derosa et al., 2016). It can also improve glucose metabolism and reverse the development of impaired glucose tolerance to diabetes (Torgerson et al., 2004). But orlistat has the risk of causing a significant reduction of fatty vitamins and rare cases of severe liver injury have also been reported (Bray and Ryan, 2012). Lorcaserin promotes satiety by selectively activating 5-HT2C receptors on opioid melanocyte precursor (POMC) neurons in the arcuate nucleus of the hypothalamus (Donnelly et al., 2009), which can effectively reduce weight (Dong et al., 2017) and cardiovascular risk factors (Marso et al., 2016). However, *in vivo* studies have found that Lorcaserin has the risk of cancers, such as colorectal cancer, pancreatic cancer, and lung cancer (Sharretts et al., 2020). Liraglutide is used for weight management in chronic diseases. It achieves weight loss and reduces cardiovascular risk factors by increasing glucose sensitivity and inhibiting glucagon production. It can also reduce liver gluconeogenesis and slow stomach transport, promoting satiety and reducing energy intake (Astrup et al., 2012). But liraglutide is contraindicated in persons with a family history of medullary thyroid cancer or type 2 of multiple endocrine neoplasia. PHEN/TPM ER can stimulate the hypothalamus to release catecholamines and inhibit the reuptake of norepinephrine (Swift et al., 2014), reducing appetite and food consumption to effective weight loss (Garvey et al., 2012). It can also improve blood pressure, blood sugar, high-density lipoprotein, triglyceride, and total cholesterol (Gadde et al., 2011; Allison et al., 2012). However, PHEN/TPM ER has a greater risk of side effects, causing kidney stones and increasing heart rate. Use in the first trimester of pregnancy can increase the risk of cleft lip and cleft palate in infants, and the drug should not be used in patients with glaucoma. The studies of naltrexone/bupropion (Greenway et al., 2010) have shown that it can effectively reduce body weight and HbA1c by inhibiting neuronal reuptake of dopamine and norepinephrine (Polak et al., 2006; Hollander et al., 2013). It also reduces waist circumference, fasting blood glucose, insulin level, high-density lipoprotein, and total cholesterol, but it will temporarily increase blood pressure and heart rate.

Surgical treatment of obesity has a definite curative effect. It is the first choice, especially for obese people with a high BMI which seriously affects their health and quality of life. At the same time, bariatric surgery also has some problems such as high perioperative and postoperative risks, certain complications, and difficulty to overcome fear.

Compared with western medicine and surgery, patients using TCM treatment for weight reduction are well tolerated and have no serious adverse effects. It applies to a wide range of people and has no clear contraindications. While reducing body weight, it can also alleviate some discomfort symptoms, such as can’t stand the heat, hyperhidrosis, stickiness in the mouth, and so on. TCM can regulate people’s body composition and thus improve their quality of life (Sang et al., 2018).

#### 4.2.2 Regulating glucose and lipid metabolism

The study found that, compared with placebo, TCM preparations treatment can reduce TG and TCHO, and improve HDL in overweight and obese patients. It can also reduce fasting blood glucose in overweight and obese patients with abnormal blood glucose. These results proved that TCM was involved in metabolic pathways *in vivo*, especially glucose and lipid metabolism. Modern pharmacology has also proved that saponins, polysaccharides, alkaloids, polyphenols, and other active ingredients in TCM can lose weight. They fight obesity by suppressing appetite, reducing digestion and absorption of exogenous lipids, and promoting oxidation and consumption of lipids (Zhang et al., 2014).

At the same time, compared with placebo, TCM treatment can also effectively reduce FBG of overweight and obese people with abnormal blood glucose. However, this article is inconclusive as to whether TCM modulates HbA1c, we can explore this further by including more studies in the future.

It can be seen that TCM was also involved in metabolic pathways in the body while exerting weight reduction effects. It takes advantage of the synergistic effect between drugs, participates in the regulation of glucose and lipid metabolism and blood pressure through multiple targets, and improves many potential metabolic risk factors associated with overweight and obesity (He et al., 2016). In addition, TCM can improve people’s body composition (Sang et al., 2018) and reduce many discomfort symptoms (Zhang and Shen, 2013). The general population has good tolerance to TCM, and no serious adverse reactions have been found.

#### 4.2.3 Probe into the potential mechanism of TCM

TCM intervention can reduce weight, correct glucose and lipid disorders, and regulate blood pressure compared to control. Its mechanism may be realized by regulating fat metabolism, intestinal flora, and hormone level, but the mechanism for muscle, liver, and pancreas is not clear (Li et al., 2020). Understanding from syndrome differentiation and treatment, Damp Heat Syndrome (DHS) is one of the most common “syndromes” in TCM (Ahirwar and Mondal, 2019), and obesity mostly belongs to “DHS”. DHS is mainly characterized by changes in inflammatory factors and abnormal immune function. It is also closely related to oxidative damage, energy metabolism, endotoxin production, blood lipid metabolism, etc., (Guo et al., 2020; Zhang G. D. et al., 2021). In addition, obesity is prone to insulin resistance, which eventually leads to serious metabolic disorders. Anoxia is an important feature of “dampness” (Lu, 2010), “dampness” can lead to circulatory disorder and anoxia of adipose tissue and the small intestine. Also, the consumption of fatty and oily or sweet foods can cause fat accumulation and internal heat (Ahirwar and Mondal, 2019). Excessive production of free fatty acids (FFA) can lead to lipotoxicity or “lung toxicity” in the TCM, they together lead to chronic low-grade systemic inflammation. TCM may act on multiple targets of the pathological pathway of “lipotoxicity (non-toxicity) - inflammation - DHS - insulin resistance - metabolic disease”. “Heat clearing” and “dampness clearing” drugs are mainly used to correct the situation of damp heat and relieve the symptoms of “heavy body trapped, fear of heat, thick and greasy tongue coating”. These herbal drugs can improve glucose and lipid metabolism, reduce inflammation *in vivo*, inhibit hypoxia inducible factor (HIF), and reverse insulin resistance, to prevent and treat obesity metabolic disorder (Zhang C. H. et al., 2021). A large number of *in vitro* studies and animal experiments show that TCM has the potential for the multi-target treatment of obesity (Li et al., 2020).

#### 4.2.4 Limitations of the study

However, these studies still have many limitations. First of all, the quality of original documents is not high, so the evidence level can be improved by enhancing the quality of TCM clinical trials in the future. Secondly, some high-quality studies of TCM for obesity and its complications are mainly yellow race. Therefore, more extensive studies are needed to clarify the practicality of TCM in different ethnic groups. Third, metabolism-related indicators such as blood pressure, blood glucose, and blood lipids are rarely included in clinical trials, with small sample sizes and large heterogeneity. Consequently, interpretation of the results needs to be cautious, and high-quality and large samples are needed to prove it. Finally, none of the included studies were followed up. It is unclear whether there is a rebound in weight loss and the long-term weight maintenance, so the long-term effects of weight loss with TCM treatment need further study.

## 5 Conclusion

In this meta-analysis of RCTs, TCM preparations can effectively reduce the weight, BMI, waist circumference, and hip circumference of overweight and obese people compared to control. Meanwhile, TCM preparations can regulate FBG and lipid metabolism and control blood pressure through multi-system treatment. However, long-term effects of TCM on weight loss still need to be further explored, which is also our future research goal.

## Data Availability

The original contributions presented in the study are included in the article/Supplementary Material, further inquiries can be directed to the corresponding authors.

## Abbreviations

ALT, alanine aminotransferase; AST, aspartate aminotransferase; BMI, body mass index; DBP, diastolic blood pressure; DHS, damp-heat syndrome; FBG, fasting blood glucose; HbA1c, glycated hemoglobin; HDL, high-density lipoprotein; IGT, Impaird glucose tolerance; LDL, low-density lipoprotein; SBP, systolic blood pressure; TCHO, Total cholesterol; TCM, Traditional Chinese Medicine; TG, Triglycerides.
